# Supplementary figures and images for: Conserved and unique features of terminal telomeric sequences in ALT-positive cancer cells
Source: eLife. 2025 Aug 1;14:RP106657. doi: 10.7554/eLife.106657 (PMC12316455; doi:10.7554/eLife.106657)

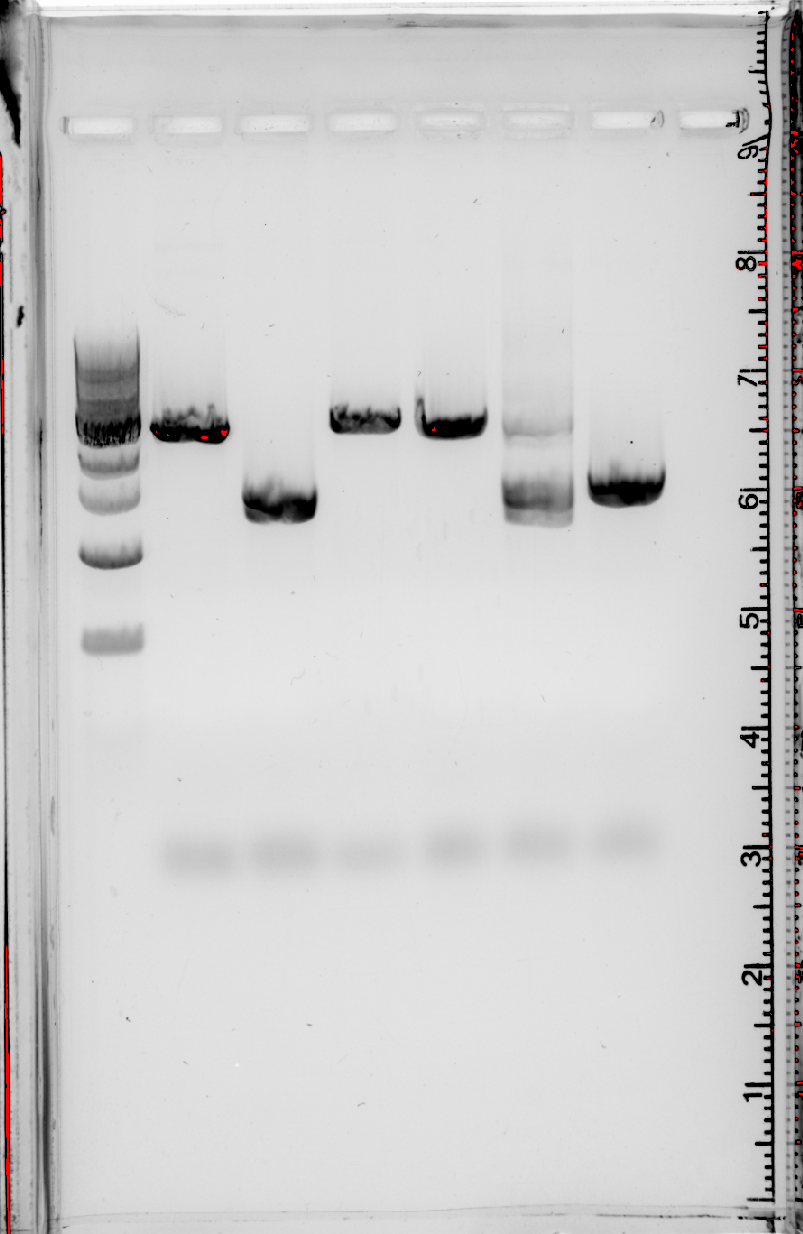

Supplement: Figure 2—source data 1. [file elife-106657-fig2-data1.zip › Fig 2_source-data/Figure 2 - supp. fig - panel B.tif]

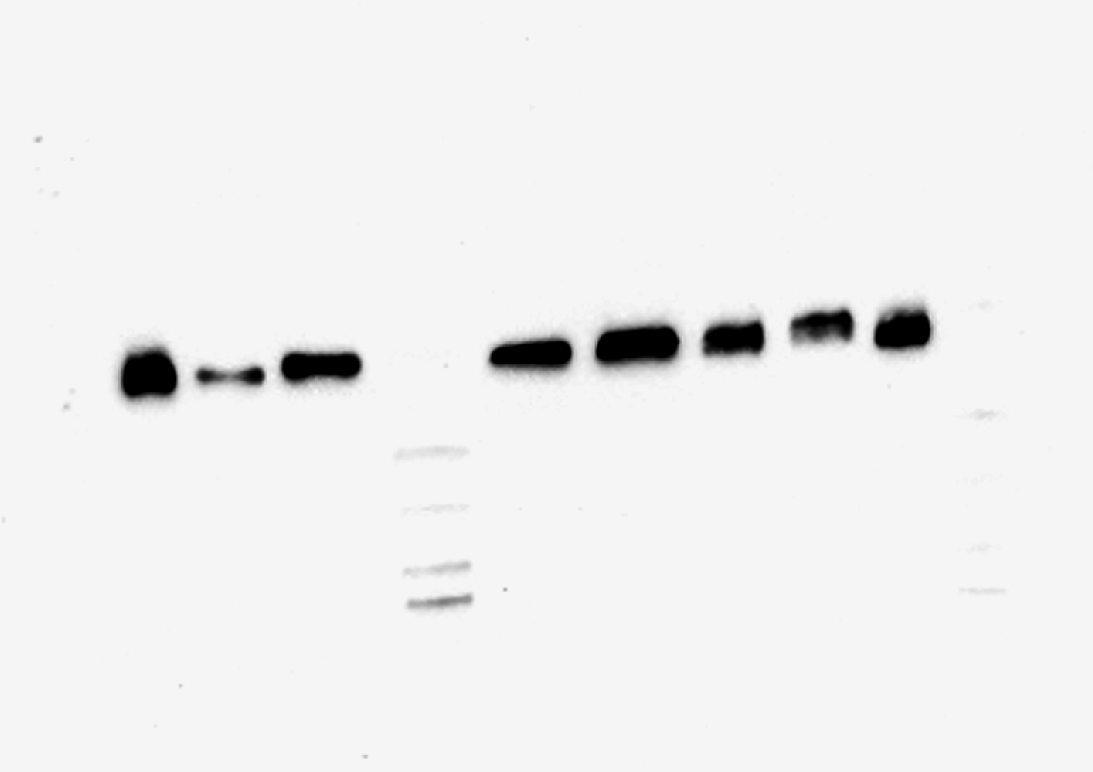

Supplement: Figure 2—source data 1. [file elife-106657-fig2-data1.zip › Fig 2_source-data/Figure 2 - supp. fig - panel C - bottom panel.tif]

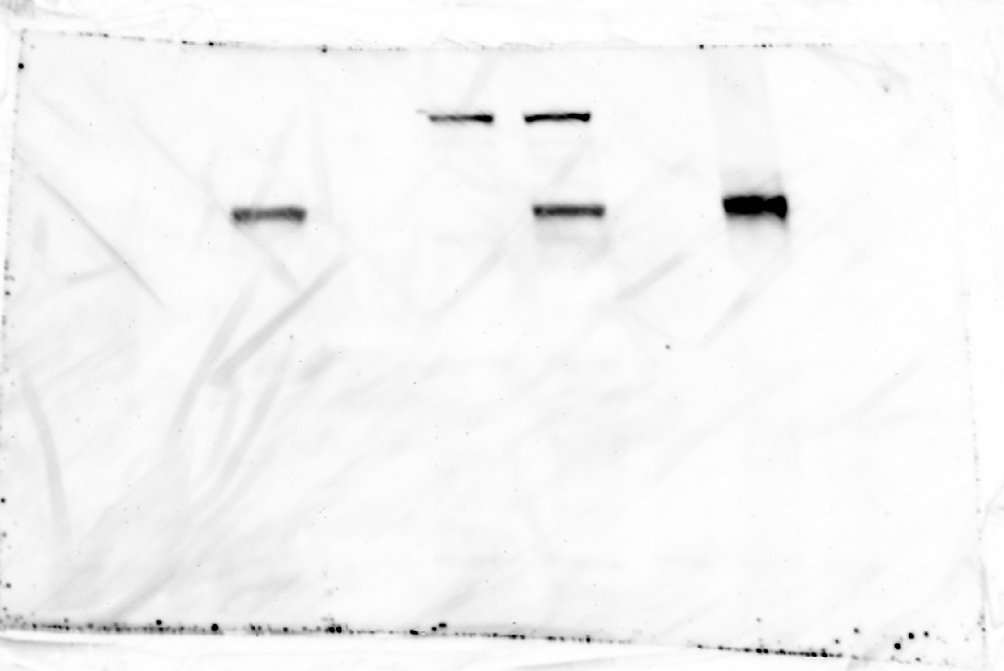

Supplement: Figure 2—source data 1. [file elife-106657-fig2-data1.zip › Fig 2_source-data/Figure 2 - supp. fig - panel C -top panel.tif]

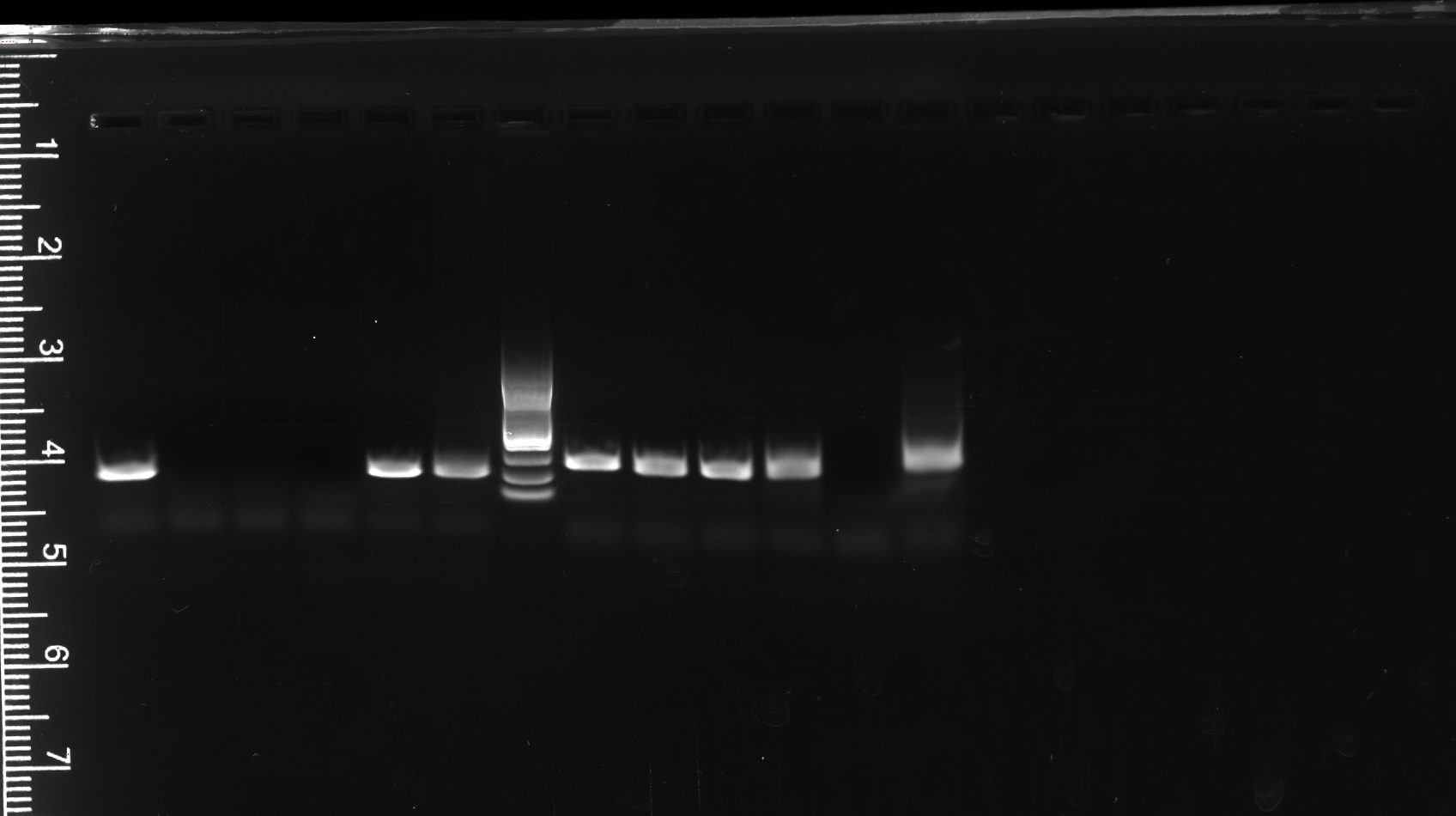

Supplement: Figure 2—source data 1. [file elife-106657-fig2-data1.zip › Fig 2_source-data/Figure 2 - supp. fig - panel E.tif]

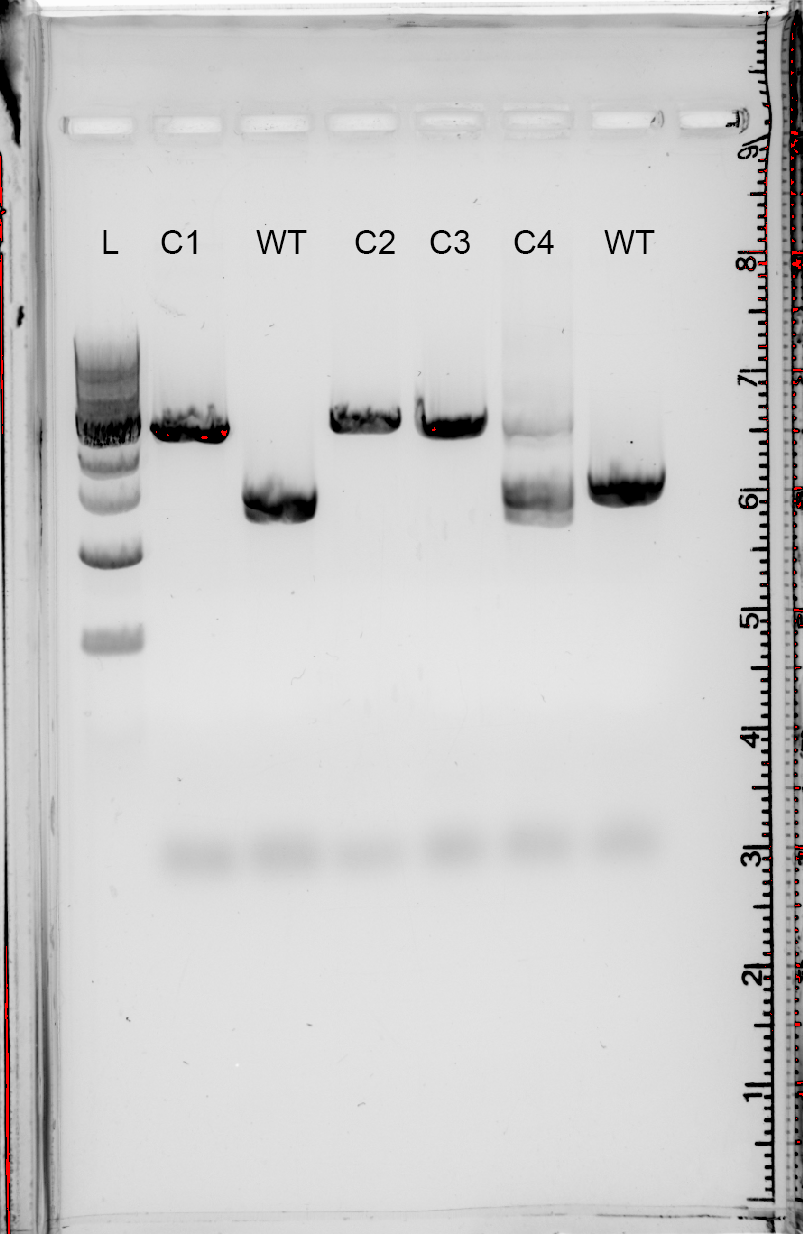

Supplement: Figure 2—source data 2. — Samples used in the panels are panel B (agarose gel): samples include wild-type (WT), clone 2 (C2), and clone 3 (C3). ‘L’ indicates a 1 kb ladder. Panel C (western blot): clone 3 (C3) with and without dTAG treatment. ‘L’ indicates Precision Plus Protein Standards. FLAG and tubulin antibodies were used for the top and bottom panels, respectively. Panel E (agarose gel): samples include clone 3 (C3), clone 4 (C4), and wild-type (WT). ‘L’ indicates a 100 bp ladder. [file elife-106657-fig2-data2.zip › Fig 2_source-data_labelled/Figure 2 - supp. fig - panel B _labelled.tif]

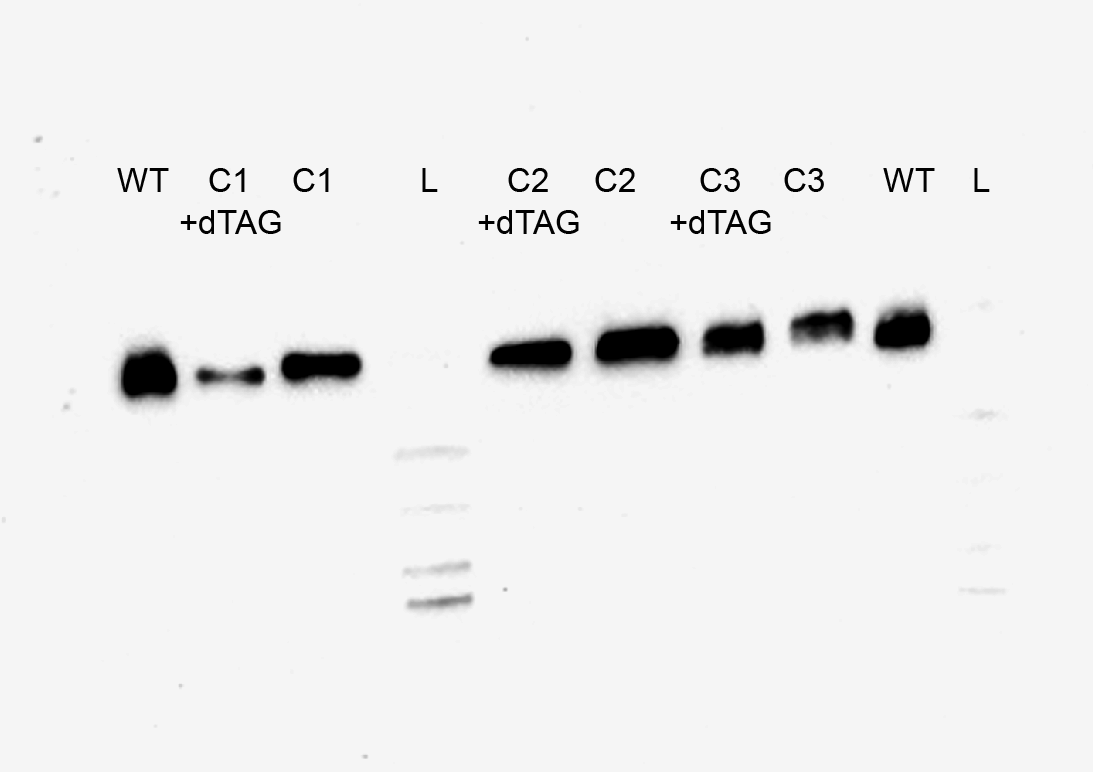

Supplement: Figure 2—source data 2. — Samples used in the panels are panel B (agarose gel): samples include wild-type (WT), clone 2 (C2), and clone 3 (C3). ‘L’ indicates a 1 kb ladder. Panel C (western blot): clone 3 (C3) with and without dTAG treatment. ‘L’ indicates Precision Plus Protein Standards. FLAG and tubulin antibodies were used for the top and bottom panels, respectively. Panel E (agarose gel): samples include clone 3 (C3), clone 4 (C4), and wild-type (WT). ‘L’ indicates a 100 bp ladder. [file elife-106657-fig2-data2.zip › Fig 2_source-data_labelled/Figure 2 - supp. fig - panel C - bottom panel_labelled.tif]

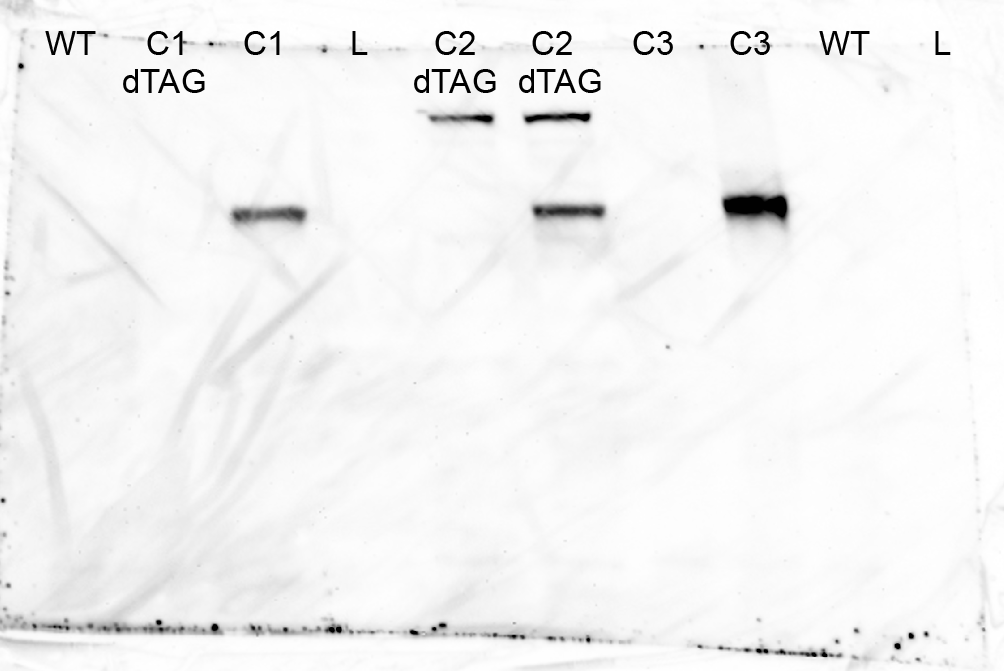

Supplement: Figure 2—source data 2. — Samples used in the panels are panel B (agarose gel): samples include wild-type (WT), clone 2 (C2), and clone 3 (C3). ‘L’ indicates a 1 kb ladder. Panel C (western blot): clone 3 (C3) with and without dTAG treatment. ‘L’ indicates Precision Plus Protein Standards. FLAG and tubulin antibodies were used for the top and bottom panels, respectively. Panel E (agarose gel): samples include clone 3 (C3), clone 4 (C4), and wild-type (WT). ‘L’ indicates a 100 bp ladder. [file elife-106657-fig2-data2.zip › Fig 2_source-data_labelled/Figure 2 - supp. fig - panel C -top panel_ labelled.tif]

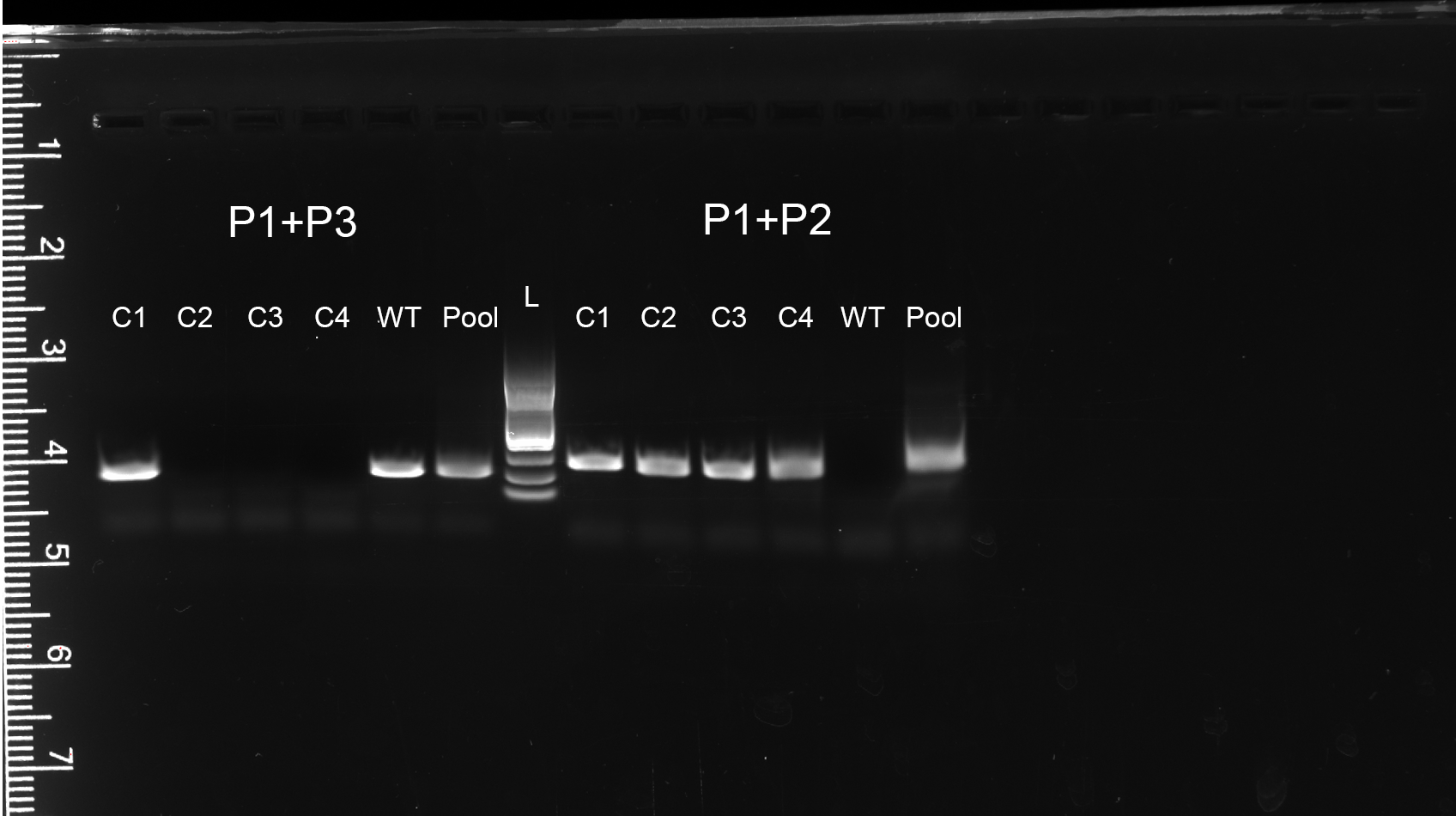

Supplement: Figure 2—source data 2. — Samples used in the panels are panel B (agarose gel): samples include wild-type (WT), clone 2 (C2), and clone 3 (C3). ‘L’ indicates a 1 kb ladder. Panel C (western blot): clone 3 (C3) with and without dTAG treatment. ‘L’ indicates Precision Plus Protein Standards. FLAG and tubulin antibodies were used for the top and bottom panels, respectively. Panel E (agarose gel): samples include clone 3 (C3), clone 4 (C4), and wild-type (WT). ‘L’ indicates a 100 bp ladder. [file elife-106657-fig2-data2.zip › Fig 2_source-data_labelled/Figure 2 - supp. fig - panel E_labelled.tif]
